# Parasitoids (Hymenoptera: Braconidae: Aphidiinae) of Northeastern Iran: Aphidiine-Aphid-Plant Associations, Key and Description of a New Species

**DOI:** 10.1673/031.012.14301

**Published:** 2012-12-09

**Authors:** Ehsan Rakhshani, Sedigheh Kazemzadeh, Petr Starý, Hossein Barahoei, Nickolas G. Kavallieratos, Aleksandar Ćetković, Anđelka Popović, lmran Bodlah, Željko Tomanović

**Affiliations:** ^1^ Department of Plant Protection, College of Agriculture, University of Zabol, 98615-538, I.R. Iran; ^2^ Laboratory of Aphidology, Institute of Entomology, Biology Centre, Academy of Sciences of the Czech Republic, Branišovská 31, 37005, České Budějovicé, Czech Republic; ^3^ Agricultural Research Institute, University of Zabol, 98615-538, I.R. Iran; ^4^ Laboratory of Agricultural Entomology, Department of Entomology and Agricultural Zoology, Benaki Phytopathological Institute, 8 Stefanou Delta str., 14561, Kifissia, Attica, Greece; ^5^ Institute of Zoology, Faculty of Biology, University of Belgrade, Studentski trg 16, 11000, Belgrade, Serbia; ^6^ Department of Entomology, Pir Mehr Ali Shah Arid Agriculture University, Rawalpindi, Pakistan

**Keywords:** *Trioxys metacarpalis* n.sp., tritrophic associations

## Abstract

Aphid parasitoids of the subfamily Aphidiinae (Hymenoptera: Braconidae) of northeastern Iran were studied in this paper. A total of 29 species are keyed and illustrated with line drawings. The aphidiines presented in this work have been reared from 42 aphid host taxa occurring on 49 plant taxa from a total of 33 sampling sites. Sixty-six aphidiine-aphid-plant associations are presented. *Trioxys metacarpalis* sp. nov. from *Chaitaphis tenuicaudata* Nevsky (Hemiptera: Aphididae) on *Kochia scoparia,* is described. The species diversity based on the comparative faunistic analysis is discussed.

## Introduction

Members of the subfamily Aphidiinae (Hymenoptera: Braconidae) are solitary endoparasitoids of aphids (Starý 1970). By being widespread and often quite abundant, they play important role in aphid population control, including the reduction of aphid pests on different crop plants (Hughes 1989; Hagvar and Hofsvang 1991; Schmidt et al. 2003; Brewer and Elliott 2004; Starý 2006). Successful use of Aphidiinae as biocontrol agents is affected by the knowledge about their taxonomy, host selection behavior, and ecology (Powell 1994; Rehman and Powell 2010).

Iran is commonly known as the cross-road between the Palaearctic and Oriental regions (Starý 1979; Starý et al. 2000). Biosystematics of the aphidiine parasitoids, including tritrophic associations, have recently been investigated in different parts of Iran (Starý et al. 2000, 2005; Bagheri-Matin et al. 2006, 2007a,b, 2008a,b,c, 2010; Rezaei et al. 2006; Tomanović et al. 2007; Barahoei et al. 2010, 2011), but there are still areas insufficiently studied, such as the northern part of Iran. The northern part is surrounded by the Alborz mountains, which separate two markedly different climatic and vegetation zones: a high rainfall zone with diversified vegetation in the north and a desert area in the south. The eastern extremity of this mountain range is connected with the highlands in neighboring Afghanistan and Turkmenistan, acting as a biogeographical corridor between highland regions with diversified faunal elements, but also representing an isolated complex in the vast, predominantly dry areas of Central Asia. This study was focused on the Khorasan e Shomali province, where the green valleys and sub-mountainous regions were principally surveyed. There are very few records of the aphid parasitoids and their trophic associations from this region (Starý 1979). The purpose of this study was to identify the spectrum of aphidiines attacking aphids feeding on plants in this area, as well as to provide information about their host range pattern. Five years of data collecting on aphidiine-aphid-plant associations are presented together with the description of a new species and a key to the aphidiine species found.

## Materials and Methods

**Figure 1.**
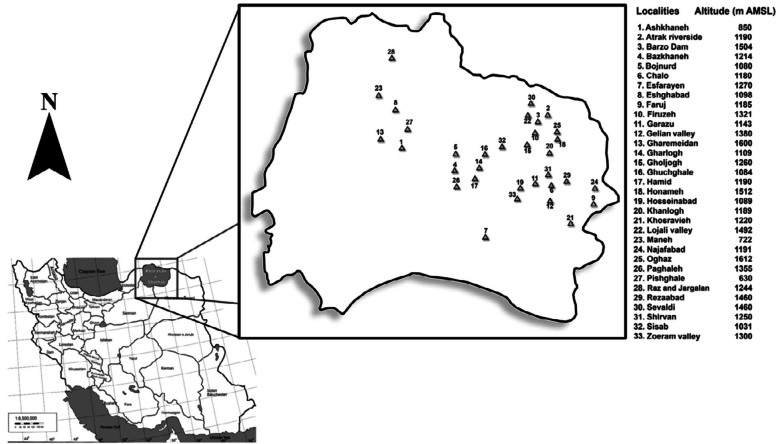
Map of the sampling localities at Khorasan e Shomali province (northeastern Iran). The numbers on the map are listed to the right of the figure along with the altitudes in meters above mean sea level. High quality figures are available online.

The studies were carried out at 33 localities in Khorasan e Shomali province (Figure 1), which is a representative lowland to the submountainous area of the northeastern part of Iran, covering an altitudinal range from 630 to 1612 meters above mean sea level. Samples were collected from 2004 to 2009. Samples from different host plants with aphid colonies were carefully cut off, subsequently put inside semi-transparent plastic boxes covered by mesh, and transferred to the laboratory. A few live aphids were killed and preserved in a solution of two parts 90% ethanol to one part 75% lactic acid (Eastop and van Emden 1972) for identification. The rearing boxes were kept in an air-conditioned room (22° C) and were inspected daily for parasitoid emergence. The parasitoids were preserved in ethanol for further determination in the laboratory. Few specimens from each sample were dissected and slide mounted in Hoyer's medium (Krantz 1978). The external morphology of parasitoids was studied using the Nikon SMZ645 and Olympus SZX9 stereomicroscopes. The ratio measurements were based on slide-mounted specimens. See Kavallieratos et al. (2001, 2005a,b, 2006) for more details about measurements. Aphid nomenclature and classification follows Remaudière and Remaudière (1997) and that of parasitoids follows Sharkey and Wharton (1997).

## Results

A total of 29 parasitoid species from 42 aphid taxa collected were identified, occurring on 49 plant species in the studied area, and we detected 66 aphidiine-aphid-plant associations.

### Parasitoid-aphid-plant associations

***Adialytus ambiguus* (Haliday)  (Figures 11, 70)**
***Material examined*.** *Sipha maydis* Passerini on *Bromus tectorum,* Gharemeidan, 14 May 2008, 15♂♂18♀♀.

***Adialytus salicaphis* (Fitch)  (Figures 12, 45, 71)**
***Material examined.*** *Chaitophorus salijaponicus* ssp. *niger* Mordvilko on *Salix alba,* Shirvan, 24 June 2008, 32♂♂54♀♀; Esfarayen, 17 May 2008, 8♂♂13♀♀. *Chaitophorus* sp. on *Populus alba,* Gelian valley, 24 May 2008, 7♂♂8♀♀ Gholjogh, 27 October 2008, 15♂♂28♀♀.

**Figures 2–10.**
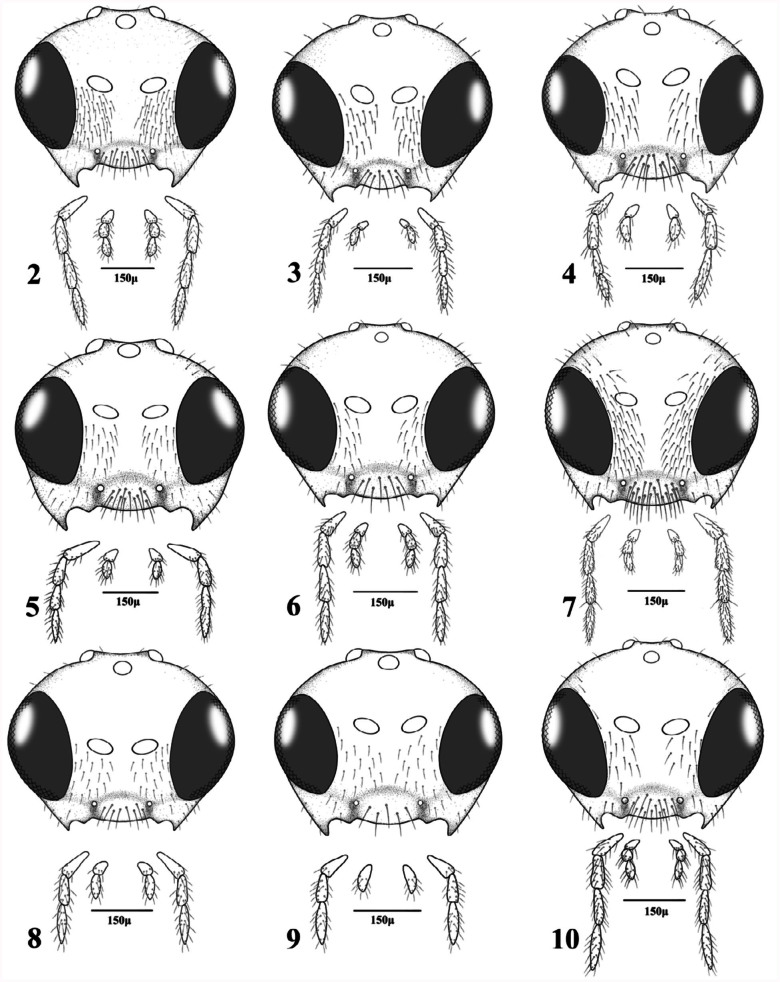
Heads and mouthparts (females). 2. *Aphidius cingulatus* Ruthe; 3. *Aphidius colemani* Viereck; 4. *Aphidius matricariae* Haliday; 5. *Aphidius persicus* Rakhshani and Starý; 6. *Aphidius transcaspicus* Telenga; 7. *Areopraon lepelleyi* (Waterston); 8. *Lysiphlebus desertorum* Starý; 9. *Lysiphlebus fabarum* (Marshall); 10. *Praon exsoletum* (Nees). High quality figures are available online.

***phidius cingulatus* Ruthe  (Figure 2, 21, 85)**
***Material examined*.** *Pterocomma pilosum* Buckton on *Salix alba,* Faruj, 17 May 2008, 1♂2♀♀; Shirvan, 23 April 2008, 1♀.

***Aphidius colemani* Viereck  (Figures 3, 13, 46, 54, 72)**
***Material examined*.** *Aphis fabae* Scopoli on *Abelmoschus esculentus,* Shirvan, 29 October 2007, 3♂♂6♀♀. *Aphis punicae* Passerini on *Punica granatum,* Shirvan, 08 September 2007, 3♀♀. *Aphis rumicis* L. on *Rumex acetosella,* Bojnurd, 01 October 2007, 15♂♂35♀♀. *Hayhurstia atriplicis* (L.) on *Chenopodium album,* Faruj, 08 November 2007, 41♂♂48♀♀.

***Aphidius ervi* Haliday  (Figures 14, 55, 73)**
***Material examined*.** *Acyrthosiphon pisum* (Harris) on *Medicago sativa,* Chalo, 09 October 2007, 1♂3♀♀; Eshghabad, 26 May 2008, 1♂2♀♀; Sisab, 20 June 2008, 3♂♂2♀♀.

***Aphidius matricariae* Haliday  (Figures 4, 15, 39, 56, 74)**
***Material examined*.** *Aphis affinis* del Guercio on *Mentha longifolia,* Shirvan, 27 May 2008, 2♀♀. *Brachycaudus helichrysi* (Kaltenbach) on *Calendula officinalis,* Bojnurd, 14 October 2008, 4♂♂20♀♀.

***Aphidius persicus* Rakhshani and Starý  (Figures 5, 16, 75)**
***Material examined*.** *Uroleucon* sp. on *Launaea arborescens,* Maneh, 10 November 2008, 1♂2♀♀.

***Aphidius popovi* Starý  (Figures 17, 76)**
***Material examined*.** *Amphorophora* *catharinae* (Nevsky) on *Rosa* sp., Faruj, 20 May 2008, 1♂2♀♀; Shirvan, 03 November 2007, 5♀♀; Bojnurd, 27 September 2008, 1♂2♀♀; Esfarayen, 04 June 2008, 3♀.

***Aphidius smithi* Sharma and Subba Rao  (Figures 18, 77)**
***Material examined*.** *Acyrthosiphon pisum* (Harris) on *Medicago sativa,* Ghuchghale, 01 November 2007, 1♂3♀♀; Shirvan, 10 June 2008, 1♂1♀; Raz and Jargalan, 13 December 2008, 2♂♂3♀♀.

***Aphidius transcaspicus* Telenga  (Figures 6, 19, 57, 78)**
***Material examined*.** *Hyalopterus pruni* (Geoffroy) on *Prunus persica,* Shirvan, 22 June 2007, 1♂4♀♀; on *Prunus armeniaca,* Esfarayen, 09 October 2007, 3♀♀.

***Aphidius uzbekistanicus* Luzhetzki  (Figures 20, 79)**
***Material examined*.** *Sitobiun avenae* (F.) on *Triticum aestivum,* Atrak riverside, 10 June 2008, 1♂3♀♀.

***Areopraon lepetteyi* (Waterston)  (Figures 7, 22, 47, 80)**
***Material examined*.** *Eriosoma lanuginosum* (Hartig) on *Ulmus carpinifolia* var. *umbraculifera,* Shirvan, 19 May 2008, 84♂51♀♀.

***Binodoxys acalephae* (Marshall)  (Figures 23, 58, 82)**
***Material examined*** *Aphis fabae* Scopoli on *Beta vulgaris,* Bojnurd, 12 October 2004, 12♂♂9♀♀. *Aphis craccivora* Koch on *Robinia pseudoacacia,* Shirvan, 12 October 2004, 4♂♂5♀♀.

***Diaeretietta rapae* (M'Intosh)  (Figures 24, 81)**
***Material examined*.** *Brevicoryne brassicae* (L.) on *Brassica napus,* Ashkhaneh, 15 May 2008, 13♂♂25♀♀; Shirvan, 08 October 2007, 34♂♂29♀♀; on *Brassica* sp., Garazu, 12 September 2007, 12♂♂19♀♀; Bojnurd, 14 November 2008, 38♂♂5♀♀. *Diuraphis noxia* (Mordvilko) on *Triticum aestivum,* Atrak riverside, 06 March 2008, 7♂♂11♀♀; Bojnurd, 12 October 2004, 10♂♂16♀♀. *Hayhurstia atriplicis* (L.) on *Chenopodium album,* Atrak riverside, 10 June 2008, 22♂♂27♀♀; Bojnurd, 13 November 2007, 14♂♂18♀♀. *Sitobion avenae* (F.) on *Triticum aestivum,* Atrak riverside, 10 June 2008, 3♂♂5♀♀.

***Ephedrus niger* Gautier, Bonnamour and Gaumont  (Figures 25, 59, 83)**
***Material examined*.** *Uroleucon sonchi* (L.) on *Sonchus asper,* Esfarayen, 04 July 2007, 1♀. *Uroleucon jaceae aeneum* Hille Ris Lambers on *Acroptilon repens,* Sevaldi, 27 April 2008, 1♂2♀♀.

***Ephedrus persicae* Froggatt  (Figures 26, 60, 84)**
***Material examined*.** *Brachycaudus* *amygdalinus* (Schouteden) on *Prunus dulcis,* Bojnurd, 10 October 2004, 2♂♂2♀♀. *Schizaphis graminum* (Rondani) on *Triticum aestivum,* Bojnurd, 10 October 2004, 8♂♂14♀♀.

***Lipolexis gracilis* Foerster (Figures 27, 61, 86)**
***Material examined*.** *Brachycaudus amygdalinus* (Schouteden) on *Amygdalus scoparia,* Paghaleh, 12 May 2008, 2♂♂.

***Lysiphlebus confusus* Tremblay and Eady  (Figure 28)**
***Material examined*.** *Aphis verbasci* Schrank on *Verbascum* sp., Sevaldi, 25 August 2007, 3♂♂95♀♀; Khanlogh, 15 September 2007, 9♀♀; Firuzeh, 01 October 2007, 1♂24♀♀; Esfarayen, 19 June 2008, 11♀♀.

***Lysiphlebus desertorum* Starý  (Figures 8, 29, 62, 87)**
***Material examined*** *Aphis* sp. on *Artemisia herba-alba,* Shirvan, 12 October 2004, 5♂♂8♀♀.

***Lysiphlebus fabarum* (Marshall)  (Figures 9, 30, 48, 63, 88)**
***Material examined*** *Aphis affinis* del Guercio on *Mentha longifolia,* Gharlogh, 15 May 2008, 4♂♂10♀♀; Zoeram valley, 01 May 2008, 5♂♂21♀♀; Gelian valley, 25 May 2008, 10♂♂18♀♀. *Aphis alexandrae* (Nevsky) on *Carthamus oxyacantha,* Shirvan, 17 July 2007, 8♂♂16♀♀. *Aphis craccivora* Koch on *Alhagi maurorum,* Shirvan, 31 August 2007, 22♂♂51♀♀; Atrak riverside, 27 April 2008, 13♂♂46♀♀; Hamid, 25 May 2008, 38♂42♀♀; on *Glycerhizzia glabra,* Bojnurd, 10 April 2008, 22♂♂71♀♀; on *Kochia scoparia,* Shirvan, 15 October 2008, 51♂♂70♀♀; Hamid, 25 June 2008, 30♂♂14♀♀; on *Medicago sativa,* Shirvan, 05 July 2008, 10♂♂9♀♀; on *Portulaca oleracea,* Atrak riverside, 16 May 2008, 1♂6♀♀. *Aphis davletshinae* Hille Ris Lambers on *Malva parviflora,* Shirvan, 05 November 2008, 4♂♂13♀♀; Bojnurd, 11 November 2008, 2♂♂7♀♀. *Aphis fabae* Scopoli on *Cirsium arvense,* Faruj, 29 April 2007, 19♀♀; on *Solanum nigrum,* Honameh, 06 May 2008, 13♀♀; Khanlogh, 01 July 2007, 8♂♂26♀♀; Rezaabad, 26 July 2007, 3♂♂10♀♀ Pishghale, 09 May 2007, 5♂♂21♀♀; Gharemeidan, 03 June 2008, 1♂12♀♀; on *Amaranthus* sp., Bojnurd, 11 November 2008, 1♂25♀♀. *Aphis gerardianae* Mordvilko on *Euphorbia aelleni,* Atrak riverside, 19 October 2007, 9♂♂24♀♀; Firuzeh, 25 October 2007, 6♀♀. *Aphis gossypii* Glover on *Mirabilis jalapa,* Shirvan, 29 October 2008, 3♀♀. *Aphis intybi* Koch on *Cichorium intybus,* Esfarayen, 04 July 2007, 14♂♂59♀♀; *Aphis euphorbicola* Rezwani and Lampel on *Euphorbia aelleni,* Honameh, 16 May 2008, 1♂12♀♀; Oghaz, 16 May 2008, 5♀♀. *Aphis* sp. on *Galium* sp., Bojnurd, 08 November 2008, 35♂♂49♀♀; on *Centaurea* sp., Shirvan, 27 May 2008, 5♀♀. *Brachyunguis harmalae* Das on *Peganum harmala,* Gharemeidan, 03 April 2008, 3♂♂6♀♀; Eshghabad, 03 April 2008, 14♀♀. *Brachycaudus tragopogonis* (Kaltenbach) on *Tragopogon graminifolius,* Shirvan, 03 July 2007, 7♂♂56♀♀; Barzo Dam, 23 June 2007, 2♂♂32♀♀; Esfarayen, 10 July 2007, 18♀♀; Khosravieh, 10 September 2007, 4♂♂13♀♀; Bazkhaneh, 05 November 2007, 2♂♂25♀♀. *Brachycaudus cardui* (L.) on *Cirsium arvense,* Shirvan, 09 May 2007, 1♂13♀♀; Esfarayen, 04 July 2007, 1♂17♀♀.

***Pauesia antennata* (Mukerji)  (Figures 31, 49, 89)**
***Material examined*.** *Pterochloroides persicae* (Cholodkovsky) on *Prunus persica,* Barzo Dam, 15 September 2007, 2♂♂7♀♀; on *Prunus dulcis,* Shirvan, 11 May 2007, 15♀♀; Paghaleh, 23 May 2008, 2♂♂17♀♀.

***Pauesia hazratbalensis* Bhagat  (Figures 32, 50, 90)**
***Material examined*.** *Cinara tujafilina* (del Guercio) on *Thuja orientalis,* Shirvan, 09 November 2008, 20♂♂25♀♀.

***Praon exsoletum* (Nees)  (Figures 10, 33, 40, 51, 64, 91)**
***Material examined. Therioaphis trifolii*** (Monell) on *Medicago sativa,* Hosseinabad, 28 May 2008, 2♂♂6♀♀.

***Praon rosaecola* Starý  (Figures 34, 41, 65, 92)**
***Material examined*** *Macrosiphum rosae* (L.) on *Rosa* sp., Shirvan, 03 November 2008, 4♀♀; Gelian valley, 04 April 2008, 1♂2♀♀; Lojali valley, 17 April 2008, 2♂♂.

***Praon volucre* (Haliday)  (Figures 35, 42, 52, 66, 93)**
***Material examined*.** *Amphorophora catharinae* (Nevsky) on *Rosa* sp., Bojnurd, 27 September 2008, 2♀♀. *Aphis affinis* del Guercio on *Mentha longifolia,* Gelian valley, 27 May 2008, 2♂♂1♀. *Aphis craccicora* Koch on *Medicago sativa,* Khanlogh, 06 October 2007, 2♂♂5♀♀. *Aphis urticata* Gmelin on *Urtica dioica,* Zoeram valley, 01 June 2008, 2♂♂. *Uroleucon sonchi* (L.) on *Sonchus asper,* Esfarayen, 04 July 2007, 2♂♂5♀♀.

***Praon yomenae* Takada  (Figures 36, 43, 53, 67, 94)**
***Material examined*.** *Uroleucon sonchi* (L.) on *Sonchus asper,* Esfarayen, 04 July 2008, 2♀♀.

***Trioxys complanatus* Quilis  (Figures 37, 68, 95)**
***Material examined*** *Therioaphis trifolii* (Monell) on *Medicago sativa,* Najafabad, 07 July 2008, 1♂♂3♀♀.

***Trioxys metacarpalis* Rakhshani and Starý sp. nov. (Figures 97–104)**
***Diagnosis.*** On the basis of morphological characters the new species is closely related to *T. parauctus* Starý, from which it differs in having distinctly shorter R1 vein (= metacarpus) (R1/stigma ratio of 0.31–0.36 instead of 0.70–0.80 in *T. parauctus*), shape of the prongs (with strongly upcurved tip instead of straight prongs in *T. parauctus*) and different number of palpomeres in maxillary and labial palps (3 and 1 instead of 4 and 2 in *T parauctus*). *T metacarpalis* also resembles *T tanaceticola* Starý, with respect to its short R1 vein and the number of maxillary and labial palpomeres, but it is clearly distinguishable from it, on the basis of the shape of apical setae of prongs (*T. metacarpalis* has a pair of short bristle-type setae whereas *T. tanaceticola* has a pair of ovoid setae at tip of prongs).
**Description.**
Female.
Head. Eye oval, slightly converging toward clypeus (Figure 97). Malar space equal to 0.11–0.13 of longitudinal eye diameter. Central part of frons with one seta at each side. Clypeus narrow with 4–5 long setae (Figure 97). Tentorial index (tentorio-ocular line/ intertentorial line) 0.33–0.35. Maxillary palp with 3 palpomeres, labial palp 1 palpomere. Antennae 11(12)-segmented, filiform, prevailingly with erect and semierect setae which are slightly shorter than the diameter of the segments (Figure 98). Flagellar segment 1 (= F1) 2.60–2.70 times as long as its median width, without longitudinal placodes. F2 2.40–2.50 times as long as its median width, with 1–2 longitudinal placodes. F1 equal or slightly longer than F2 (Figure 98).
  Mesosoma. Mesonotum with notauli distinct only anteriorly with one row of 4–5 setae at each side (Figure 99). Propodeum smooth with two roundly divergent carinae at lower part (Figure 101). Upper part with two rows of 5-6 long setae at each side. Lower part with a single long seta below spiracles at each side.
  Forewing. Stigma triangular with almost straight anterior outline, 2.35–2.40 times as long as its width and 2.80–3.20 as long as R1 vein (Figure 100). Setae on fringe very long.
  Metasoma. Petiole short, 1.80–2.00 times as long as wide at spiracles with three long setae below the prominent spiracular tubercles and a single seta at posterio-dorsal area at each side. Ovipositor sheath sub-quadrate at base with 1–2 long and several short setae on ventral margin (Figure 103). Prongs strongly curved upwards at tip with three long perpendicular setae in the last 2/3 of the dorsal surface and 2–3 somewhat shorter setae in the proximal region of the dorsal surface. Lateral and ventral surface with 6–7 short setae. Apex of prongs semiglobular dorsally, with a pair of short setae apically (Figure 104). Ovipositor sheath elongate, 1.90–2.10 times as long as its maximal width (at base) and 3.10–3.20 times as long as its minimal width (at tip) (Figure 103).
  Color. Body generally brown. Mouthparts yellow except black tip of the mandible. Eyes and ocelli black. Remaining parts of the head brown. Antenna brown. Pedicel, F1 and F2 light brown. Legs yellowish-brown. Femur and mid part of tibiae darker. Petiole light brown. Other metasomal segments gradually darkening toward tip. Ovipositor sheath and prongs brown.
  Body length. 1.4–1.6 mm.
  Male. Unknown.
***Material examined*.** Holotype ♀, Iran: Bojnurd, 1080 m a.m.s.l., 29 September 2007, reared from *Chaitaphis tenuicauda* Nevsky on *Kochia scoparia,* leg. S. Kazemzadeh. The holotype (slide mounted) and 1♀ paratype (same data as holotype) were deposited in the collection of P. Starý (Academy of Sciences of the Czech Republic). Two ♀♀ paratypes (slide mounted) were deposited in the collection of E. Rakhshani (University of Zabol).
***Etymology*.** The name of the new species is derived from its forewing metacarpus, which is strongly reduced in length.

***Trioxys pallidus* (Haliday)  (Figures 38, 44, 69, 96)**
*Chromaphis juglandicola* (Kaltenb ach) on *Juglans regia,* Gholjogh, 08 May 2008, 4♂♂9♀♀; Firuzeh, 16 May 2008, 2♂♂4♀♀; Bazkhaneh, 25 May 2008, 3♂♂7♀♀; Esfarayen, 28 May 2008, 5♂♂8♀♀.

**Key to female aphidiines in northeastern Iran**

**1.** Forewing venation with eight cells. Forewing 3RS reaching the margin of wing (Figures 25, 26) 2
— Forewing venation with < 8 cells. Forewing RS (Figures 13–20, 21, 28–32) or r&RS (Figures 11, 12, 22–24, 27, 33–38, 100) not reaching the wing margin 3

**2.** Forewing 2RS equal or slightly shorter than 3RSa (Figure 25). Length of petiole more than 1.8 times as long as its width (Figure 59). Ovipositor sheath long and slender (Figure 83) *Ephedrus niger*

— Forewing 2RS longer than 3RSa (Figure 26). Length of petiole less than 1.5 times as long as its width (Figure 60). Ovipositor sheath short, wide toward base (Figure 84) *Ephedrus persicae*

**3.** RS+M vein present (Figures 22, 33–36). Notauli complete (Figures 40–43) 4
RS+M vein absent (Figures 11–20, 21, 23, 24, 27–32). Notauli incomplete or absent (Figures 39, 44) 8

**4.** Antennae 13–14-segmented. Propodeum areolated (Figure 47). Ovipositor sheath densely setose at apical half (Figure 80). Face, frons, and vertex densely setose in lateral margins (Figure 7) *Areopraon lepelleyi*

— Antennae 16–19 segmented. Propodeum smooth (Figures 51–53). Ovipositor sheath sparsely setose (Figures 91–94). Face, frons and vertex sparsely setose in lateral margins (Figure 10) 5

**5.** Fl dark, yellowish at base. Forewing m-cu vein tubular throughout (Figures 34, 35). Lateral mesonotal lobes pubescent or with small hairless areas (Figures 41, 42) 6
— F1 entirely yellow. Forewing m-cu vein nebulous throughout (Figure 36) or tubular at posterior part (Figure 33). Lateral mesonotal lobes with large hairless areas (Figure 40, 43) 7

**6.** Antennae (15) 16–17-segmented. Forewing stigma 1.9–2.35 times as long as R1 (Figure 34). Petiole sparsely setose (Figure 65). Ovipositor sheath with almost straight dorsal outline (Figure 92) *Praon rosaecola*

— Antennae 17–18-segmented. Forewing stigma 1.40–1.65 times as long as R1 (Figure 35). Petiole densely setose (Figure 66). Ovipositor sheath with concave dorsal outline (Figure 93) *Praon volucre*

**7.** Propodeum densely pubescent (Figure 53). Petiole with few setae (Figure 67). Ovipositor with concave dorsal outline (Figure 94) *Praon yomenae*

— Propodeum with few setae (Figure 51). Petiole sparsely setose (Figure 64). Ovipositor sheath with straight dorsal outline (Figure 91) *Praon exsoletum*

**8.** Forewing M&m-cu vein present, complete (Figures 13–20, 21, 31, 32) or incomplete (Figures 28–30) 9
— Forewing M&m-cu vein absent (Figures 11, 12, 23, 24, 37, 38) 22

**9.** Forewing M&m-cu vein complete (Figures 13–20, 21, 31, 32). Propodeum with complete central areola (Figures 46, 49, 50). Ovipositor sheath truncated at tip (Figures 72–79, 85, 89) or with lateral spatula (Figure 90) 10
— Forewing M&m-cu vein incomplete (Figures 28–30). Propodeum smooth with two divergent carinae at lower part (Figure 48). Ovipositor sheath pointed at tip (Figures 87, 88) 20

**10.** Propodeum with wide pentagonal areola (Figures 49, 50) 11
— Propodeum with narrow pentagonal areola (Figure 46) 12

**11.** Ovipositor sheath pointed at tip with strong lateral spatula bearing 4–5 long setae (Figure 90). Forewing R1 0.6–0.7 times as long as stigma (Figure 32) *Pauesia hazratbalensis*

— Ovipositor sheath truncated at tip without spatula (Figure 89). Forewing R1 1.0– 1.1 times as long as stigma (Figure 31) *Pauesia antennata*

**12.** Tentorial index 0.60–0.80. Face densely pubescent (Figure 2) *Aphidius cingulatus*

— Tentorial index less than 0.6. Face moderately pubescent (Figures 3–6) 13
13. Anterolateral area of petiole rugose (Figure 55) *Aphidius ervi*

— Anterolateral area of petiole costate (Figures 54, 57) or costulate (Figure 56) 14

**14.** Anterolateral area of petiole costate (Figures 54, 57) 15
— Anterolateral area of petiole costulate (Figure 56) 16

**15.** Antenna 16–17-segmented. Forewing R1 0.5 times as long as stigma (Figure 19) *Aphidius transcaspicus*

— Antenna 15-segmented. Forewing R1 0.8–0.9 times as long as stigma (Figure 13) *Aphidius colemani*

**16.** Labial palps with two palpomeres (Figures 4, 5) 17
— Labial palps with three palpomeres 19
17. Forewing R1 0.1–0.2 times as long as stigma (Figure 17) *Aphidius popovi*

— Forewing R1 0.4–1.0 times as long as stigma 18

**18.** Antennae 14–15-segmented. Forewing R1 0.8–1.0 times as long as stigma (Figure 15). Ovipositor sheath short (Figure 74) *Aphidius matricariae*

— Antennae 16–17-segmented. Forewing R1 0.4–0.5 times as long as stigma (Figure 16). Ovipositor sheath elongated (Figure 75) *Aphidius persicus*

**19.** Antennae 16–17-segmented *Aphidius uzbekistanicus*

— Antennae 19–21-segmented *Aphidius smithi*

20. Forewing R1 distinctly shorter than stigma (Figure 29). Petiole narrowly triangular (Figure 62). Labial palps with two palpomeres (Figure 8). Ovipositor sheath convex dorsally (Figure 87) *Lysiphlebus desertorum*

*—* Forewing R1 distinctly longer than stigma (Figures 28, 30). Petiole widely triangular (Figure 63). Labial palps with one palpomere (Figure 9). Ovipositor sheath weakly convex dorsally (Figure 88) 21

**21.** Setae on fringe of forewing similar to those on surface or shorter (Figure 30) *Lysiphlebus fabarum*

— Setae on fringe of forewing longer than those on surface (Figure 28) *Lysiphlebus confusus*

**22** Hypopygium without prongs (Figures 70, 71, 81, 86) 23
— Hypopygium with prongs (Figures 82, 95,96, 103) 26

**23.** Ovipositor sheath curved downwards (Figure 86) *Lipolexis gracilis*

— Ovipositor sheath curved upwards (Figures 70, 71, 81) 24

**24.** Ovipositor sheath truncated at tip (Figure 81). Propodeum with narrow pentagonal areola. Antennae 14–15segmented. Labial palps with two palpomeres *Diaeretiella rapae*

— Ovipositor sheath pointed at tip (Figure 70, 71). Propodeum smooth or with two short divergent carinae at lower part (Figure 45). Antennae 12–13-segmented. Labial palps with one palpomere 25

**Figures 11–20.**
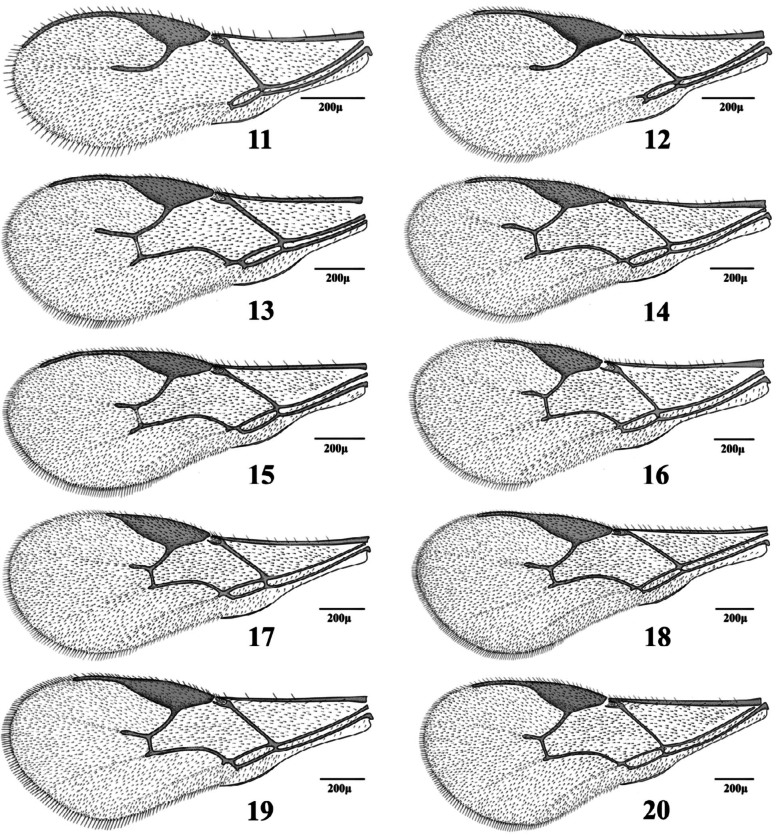
Forewings (females). 11. *Adialytus ambiguus* (Haliday); 12. *Adialytus salicaphis* (Fitch); 13. *Aphidius colemani* Viereck; 14. *Aphidius ervi* Haliday; 15. *Aphidius matricariae* Haliday; 16. *Aphidius persicus* Rakhshani and Starý; 17. *Aphidius popovi* Starý; 18. *Aphidius smithi* Sharma and Subba Rao; 19. *Aphidius transcaspicus* Telenga; 20. *Aphidius uzbekistanicus* Luzhetzki. High quality figures are available online.

**25.** R1 distinctly longer than stigma (Figure 11) *Adialytus ambiguus*

— R1 as long as or slightly shorter than stigma (Figure 12) *Adialytus salicaphis*

**26.** Petiole with primary and secondary tubercles (Figure 58) *Binodoxys acalephae*

— Petiole with only primary tubercles (Figures 68, 69, 102) 27

**27.** Prongs strongly curved upwards, bearing a pair of short setae at tip (Figure 103). R1 0.31–0.36 times as long as stigma (Figure 100) *Trioxys metacarpalis* **sp. nov.**

— Prongs almost straight, slightly curved upwards with a single claw shaped bristle at apex (Figures 95, 96). R1 0.50–0.80 times as long as stigma (Figures 37, 38) 28

**28.** Ovipositor sheath 2.10–2.20 times as long as its maximum width at base (Figure 95). R1 0.60–0.80 times as long as stigma (Figure 37) *Trioxys complanatus*

— Ovipositor sheath 2.90–3.10 times as long as it maximum width at base (Figure 96). R1 as half as stigma (Figure 38) *Trioxys pallidus*

## Discussion

The established set of the aphidiines revealed in the present contribution is dominated by the faunistic complex of Eurasian steppes (Starý 1970; Kavallieratos et al. 2004). Other faunal groups are very poorly represented, partly due to the restricted scope of the altitudinal range and the respective habitat types covered with this survey. The most noteworthy from the faunistical point is the presence of the new species, which is endemic for the province studied.

Aphid parasitoids have variable habitat preferences, which affect their trophic associations and host range patterns. They include both strictly host-specific and broadly oligophagous species (Starý 1970). Their trophic ecology and behavioral traits clearly affect their geographical distribution and phenology (Starý and Havelka 2008). In the studied area, both strictly and broadly oligophagous aphidiines were recorded. *L. fabarum* is the best example of a broadly oligophagous parasitoid that commonly parasitizes numerous aphid species of the genera *Aphis* and *Brachycaudus* on different host plants, including economically important crops. It has a wide distribution throughout Iran (Rakhshani et al. 2005a,b; Talebi et al. 2009; Mossadegh et al. 2011), including the studied area. Although there were indications that *L. confusus* is conspecific with *L. fabarum* (Belshaw et al. 1999; Carver and Franzmann 2001), *L. confusus* is still retained as a valid species because of its complex biology and the ongoing research on the revision of the genus *Lysiphlebus.*

*A. colemani* is a common parasitoid of different aphids in Iran (Rakhshani et al. 2008b,c) and other countries (Starý 1975; Kavallieratos et al. 2004, 2010), but its taxonomic status is still considered problematic, due to character overlap with *A. transcaspicus* (Mescheloff and Rosen 1990; Takada 1998; Kavallieratos and Lykouressis 1999). The host range pattern is supposed to be the important biological border between the two related species. Generally, *A. transcaspicus* is commonly associated with the genera *Hyalopterus* and *Melanaphis,* while *A. colemani* manifests a wide range of host aphids, excluding the above mentioned associations. Furthermore, some samples reared from *R padi* in Turkey (Asian et al. 2004), and *Phorodon humuli* (Schrank) or *Rhopalosiphum nymphae* (L.) in Iran (Starý et al. 2000), yielded an *“A. transcaspicus*-like” species whose identity is the matter of further research.

*L. gracilis* is newly recorded from the eastern part of Iran, after first being detected in northwestern Iran (Rakhshani et al. 2008a). It has been previously established in the areas near to northwestern and southwestern Iran, such as Georgia (Achvlediani 1981), Turkey (Rakhshani et al. 2008a), and Pakistan (Starý et al. 1998). Thus, it was expected to be present in the northeastern Iran, as was confirmed by the present study.

In the studied area *D. rapae* was recorded from *B. brassicae,* its common host, on *Brassica* spp. but also from aphids on cereals (i.e., *S. avenae, D. noxia*) and weeds (i.e., *H. atriplicis*). Wide trophic diversity of this parasitoid has been also observed in southeastern Europe (Kavallieratos et al. 2004).

The presence of the rare and strictly oligophagous parasitoids *A. lepelleyi, P. antennata,* and *P. hazratbalensis* complements the functional diversity of trophic interactions within the parasitoid complexes in the sampled area. *Areopraon* is a small aphidiine genus with a poorly known biology (Starý 1976). *A. lepelleyi* has recently been collected as a new faunal record from Iran and central Asia (Kazemzadeh et al. 2009). *Pauesia hazratbalensis* Bhagat was recorded from Iran as a rare lowland species in association with *C. tujafilina* (Starý et al. 2005). On the basis of a very characteristic spatulated ovipositor sheath, the species was placed previously into the distinct subgenus *Kashmirpauesia* (Bhagat 1981), but the validity of generic subdivision was disputed by Sanchis et al. (2001). However, it may still be of interest to investigate possible relationships of this species to other representatives with spatulated ovipositor sheaths from the Russian Far East.

The newly described species, *T. metacarpalis,* was reared from a very specific aphid-plant association, *C. tenuicauda - K scoparia.* There was confusion in the past regarding the generic identity of the host aphid associated with *Trioxys chaitaphidis* Mackauer. This parasitoid was described from the supposed *Chaitaphis* sp. aphid (Mackauer 1962), which later turned out to be the misidentified *Coloradoa heinzei* (Börner) on *Artemisia austriaca* as host-plant (Mackauer and Starý 1967). For this reason, a quite different name (*metacarpalis*) was given to the new species associated with the original *Chaitaphis* host. *T. metacarpalis* has a very short metacarpus in the forewing, a shared synapomorphy with some other species of *Trioxys,* such as *Trioxys pannonicus* Starý and *Trioxys tanaceticola* Starý. The reductions in number of maxillary and labial palpomeres are additional synapomorphy of possible significance in interpretation of their evolutionary relationships. Further morphological and molecular analyses with special emphasis on biological characteristics are needed to elucidate the phylogenetic position of the new species and the group of allied members within the genus *Trioxys.*

**Figures 21–30.**
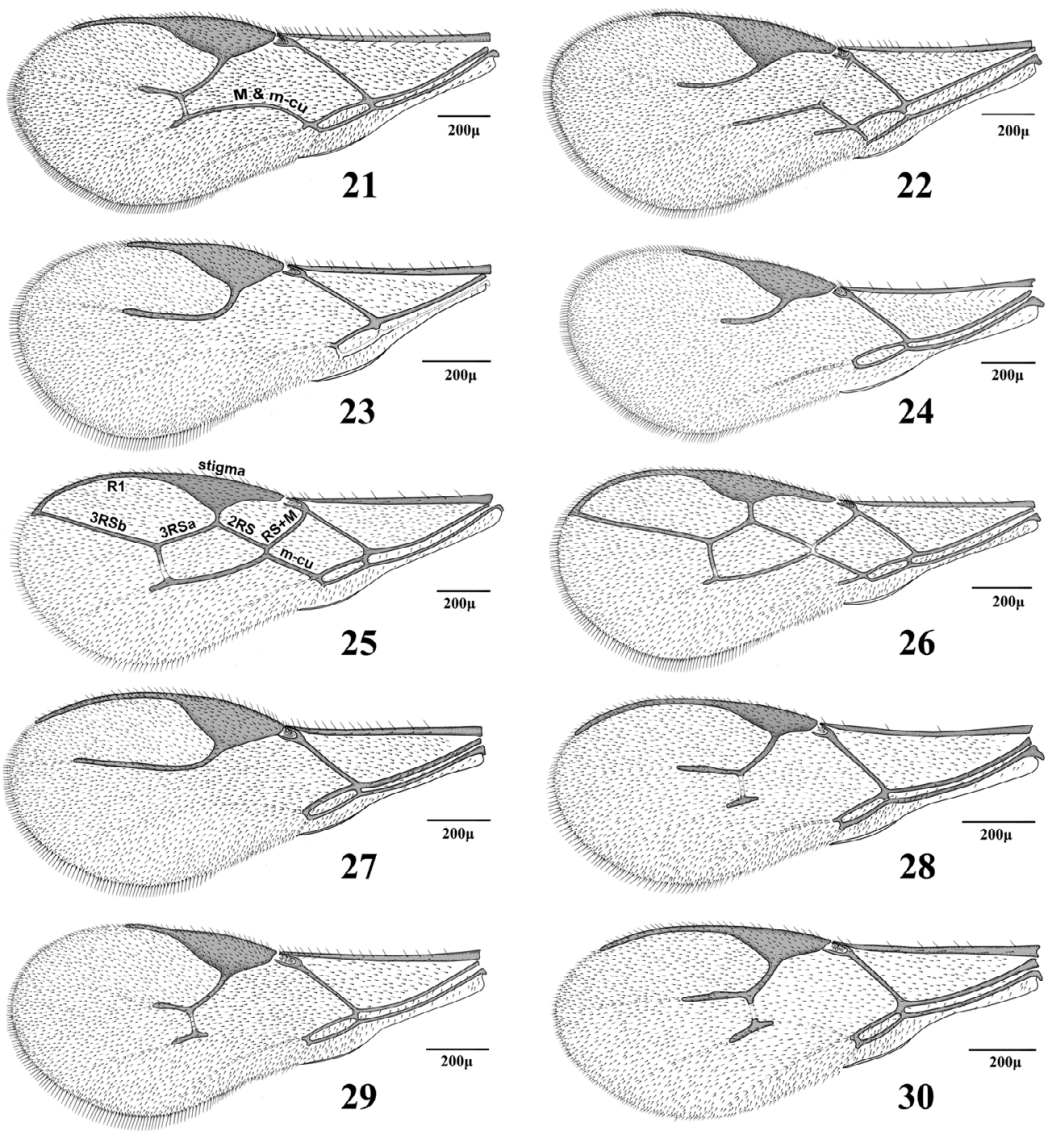
Forewing (females). 21. *Aphidius cingulatus* Ruthe; 22. *Areopraon lepelleyi* (Waterston); 23. *Binodoxys acalephae* (Marshall); 24. *Diaeretiella rapae* (M'Intosh); 25. *Ephedrus niger* Gautier, Bonnamour and Gaumont; 26. *Ephedrus persicae* Froggatt; 27. *Lipolexis gracilis* Foerster; 28. *Lysiphlebus confusus* Tremblay and Eady; 29. *Lysiphlebus desertorum* Starý; 30. *Lysiphlebus fabarum* (Marshall). High quality figures are available online.

**Figures 31–38.**
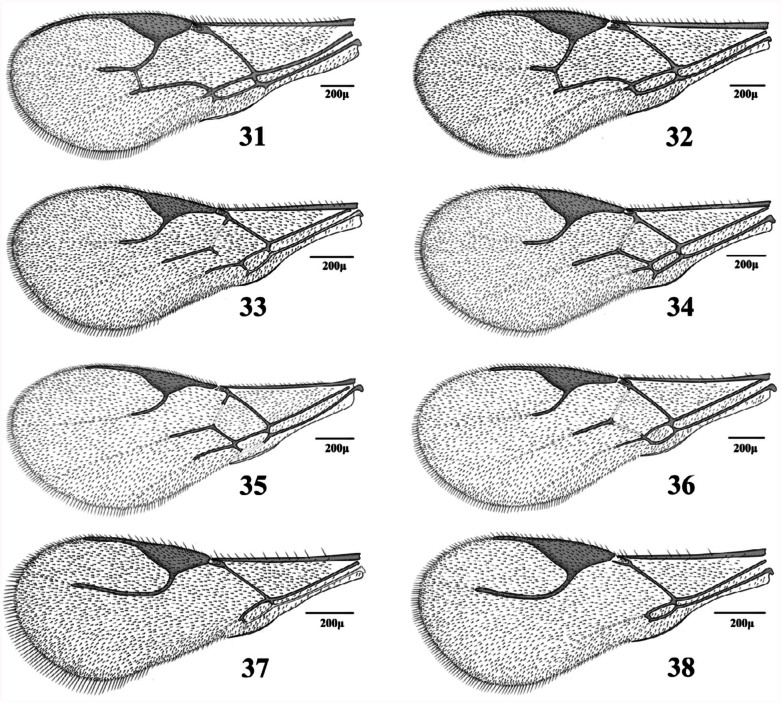
Forewing (females). 31. *Pauesia antennata* (Mukerji); 32. *Pauesia hazratbalensis* Bhagat; 33. *Praon exsoletum* (Nees); 34. *Praon rosaecola* Starý; 35. *Praon volucre* (Haliday); 36. *Praon yomenae* Takada; 37. *Trioxys complanatus* Quilis; 38. *Trioxys pallidus* (Haliday). High quality figures are available online.

**Figures 39–44.**
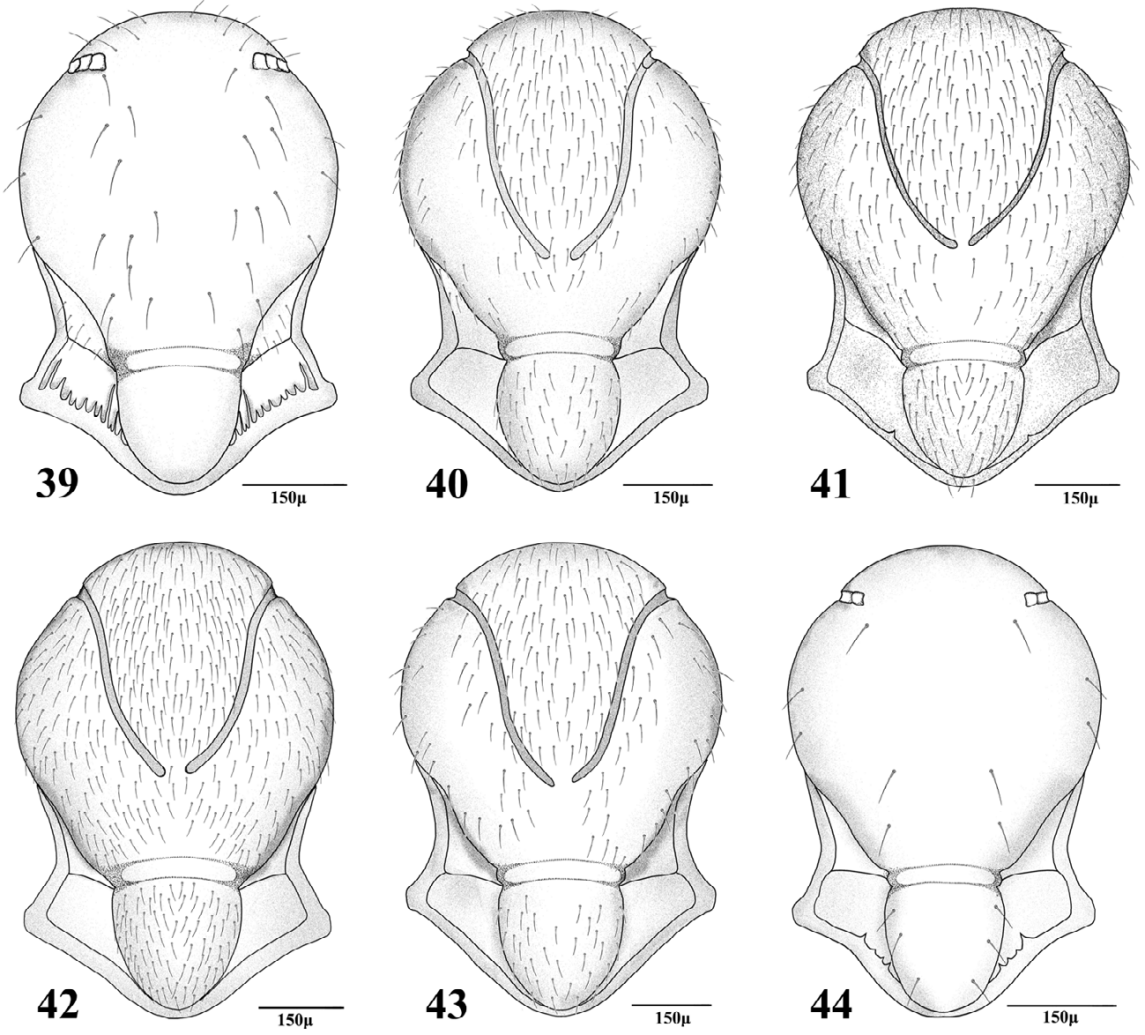
Mesonotum (females). 39. *Aphidius matricariae* Haliday; 40. *Praon exsoletum* (Nees); 41. *Praon rosaecola* Starý; 42. *Praon volucre* (Haliday); 43. *Praon yomenae* Takada; 44. *Trioxys pallidus* (Haliday). High quality figures are available online.

**Figures 45–53.**
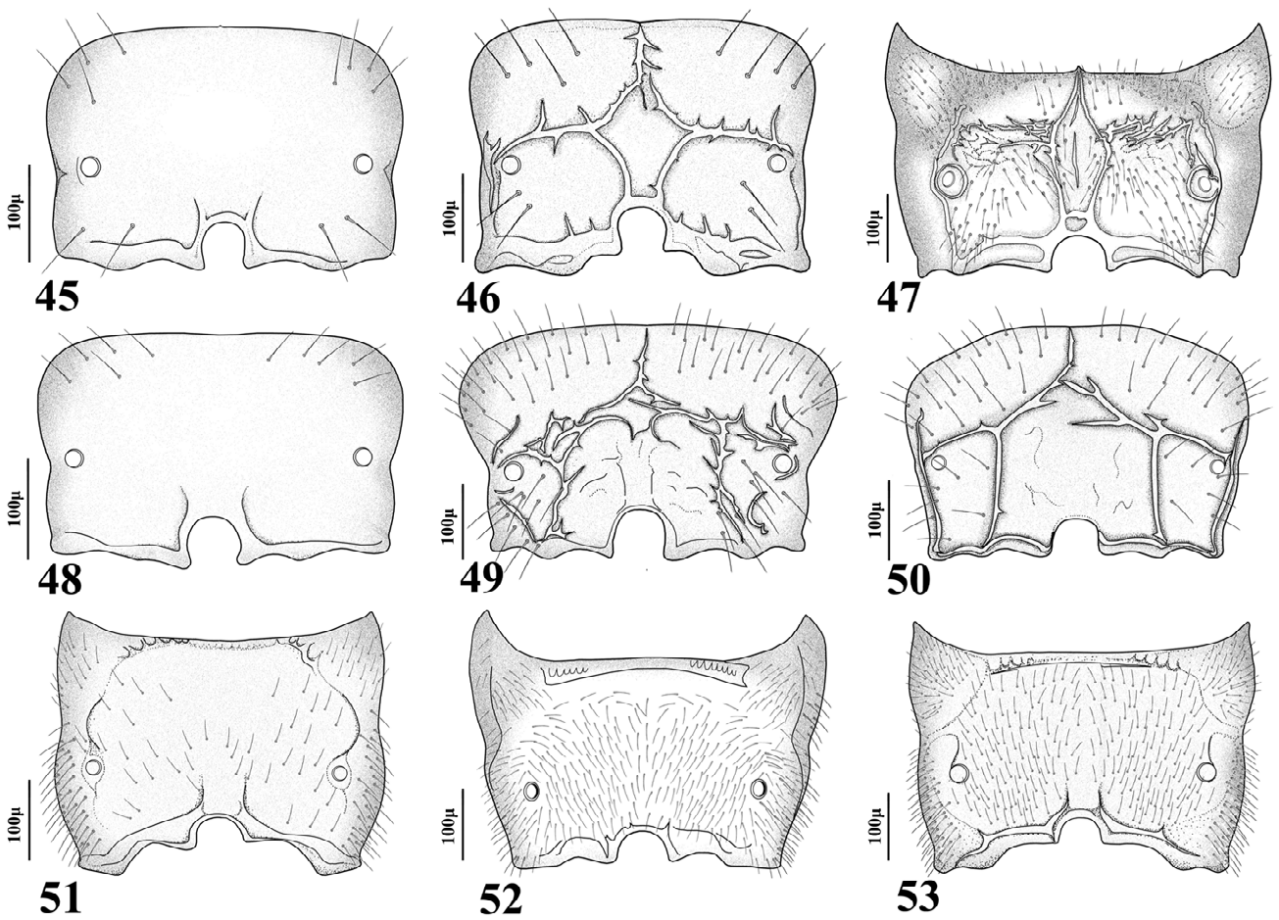
Propodeum (females). 45. *Adialytus salicaphis* (Fitch); 46. *Aphidius colemani* Viereck; 47. *Areopraon lepelleyi* (Waterston); 48. *Lysiphlebus fabarum* (Marshall); 49. *Pauesia antennata* (Mukerji); 50. *Pauesia hazratbalensis* Bhagat; 51. *Praon exsoletum* (Nees); 52. *Praon volucre* (Haliday); 53. *Praon yomenae* Takada. High quality figures are available online.

**Figures 54–69.**
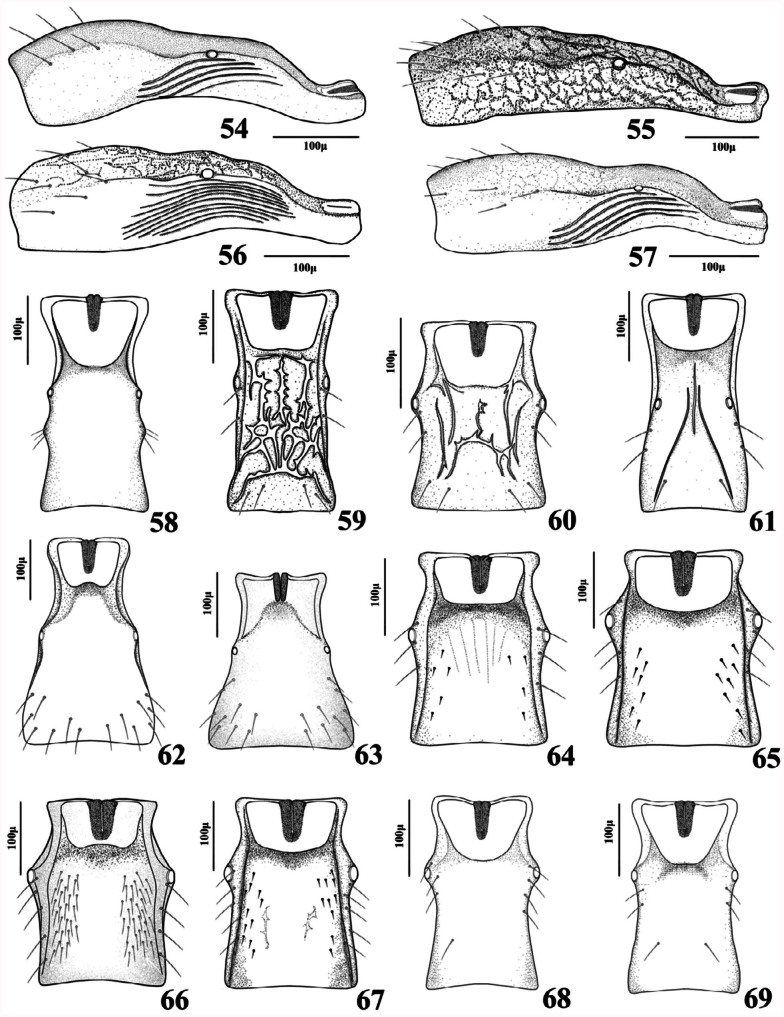
Lateral and dorsal aspect of petiole (females). 54. *Aphidius colemani* Viereck; 55. *Aphidius ervi* Haliday; 56. *Aphidius matricariae* Haliday; 57. *Aphidius transcaspicus* Telenga; 58. *Binodoxys acalephae* (Marshall); 59. *Ephedrus niger* Gautier, Bonnamour and Gaumont; 60. *Ephedrus persicae* Froggatt; 61. *Lipolexis gracilis* Foerster; 62. *Lysiphlebus desertorum* Starý; 63. *Lysiphlebus fabarum* (Marshall); 64. *Praon exsoletum* (Nees); 65. *Praon rosaecola* Starý; 66. *Praon volucre* (Haliday); 67. *Praon yomenae* Takada; 68. *Trioxys complanatus* Quilis; 69. *Trioxys pallidus* (Haliday). High quality figures are available online.

**Figures 70–84.**
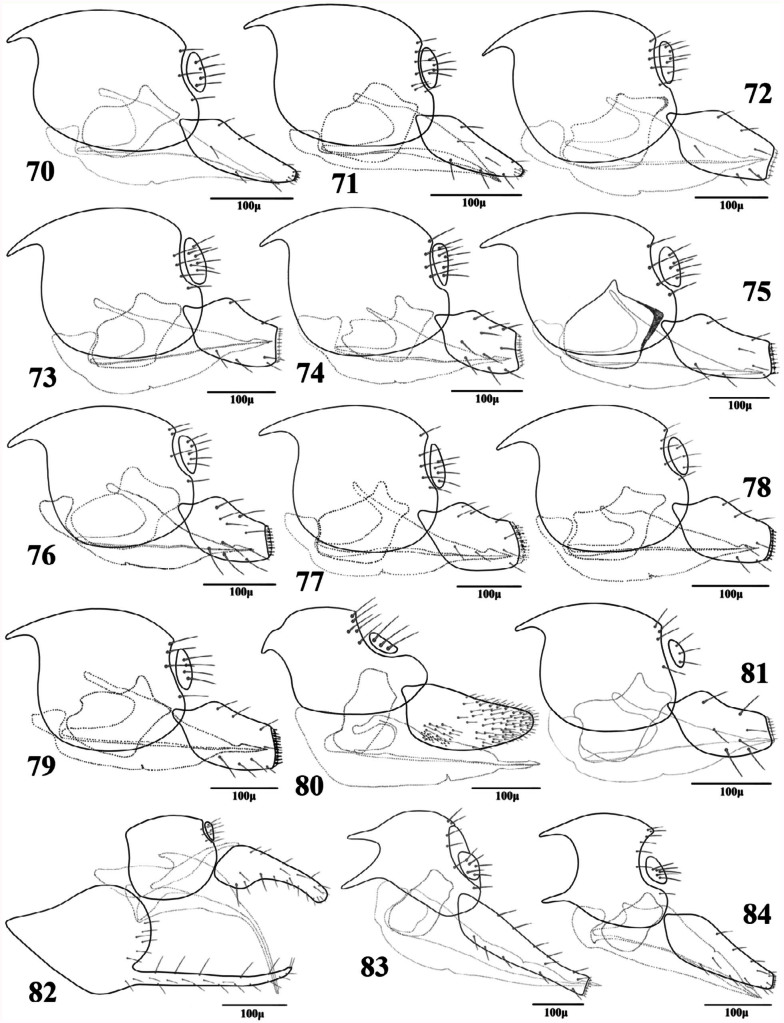
Lateral aspect of genitalia (females). 70. *Adialytus ambiguus* (Haliday); 71. *Adialytus salicaphis* (Fitch); 72. *Aphidius colemani* Viereck; 73. *Aphidius ervi* Haliday; 74. *Aphidius matricariae* Haliday; 75. *Aphidius persicus* Rakhshani and Starý; 76. *Aphidius popovi* Starý; 77. *Aphidius smithi* Sharma and Subba Rao; 78. *Aphidius transcaspicus* Telenga; 79. *Aphidius uzbekistanicus* Luzhetzki; 80. *Areopraon lepelleyi* (Waterston); 81. *Diaeretiella rapae* (M'lntosh); 82. *Binodoxys acalephae* (Marshall); 83. *Ephedrus niger* Gautier, Bonnamour and Gaumont; 84. *Ephedrus persicae* Froggatt. High quality figures are available online.

**Figures 85–96.**
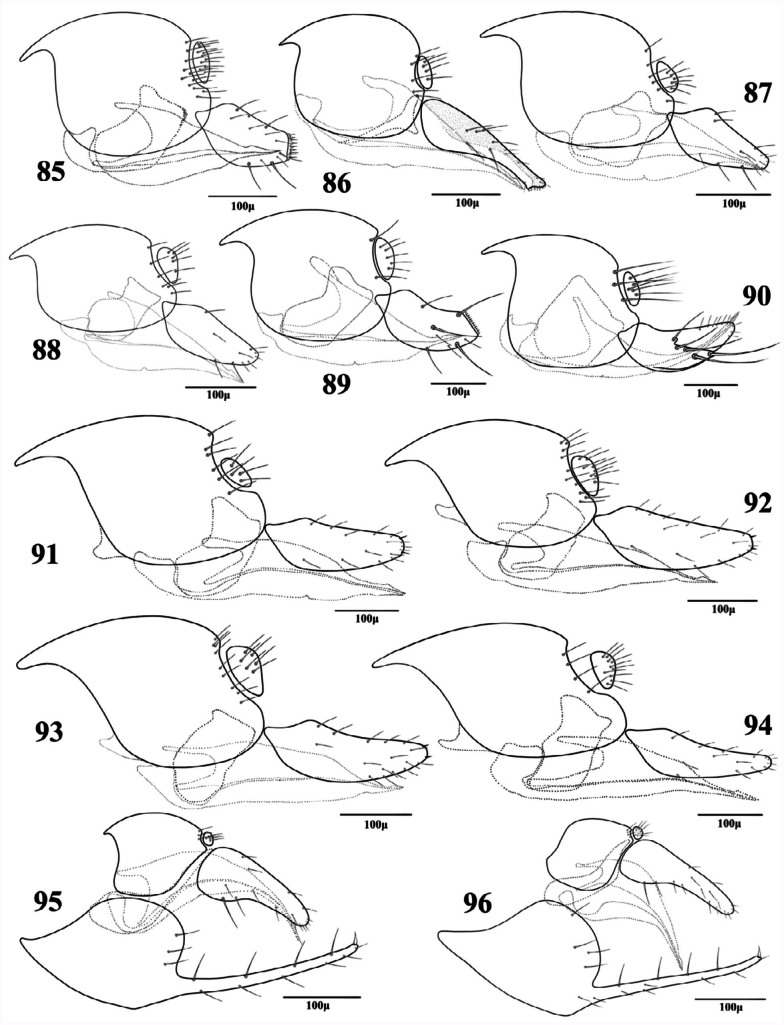
Lateral aspect of genitalia (females). 85. *Aphidius cingulatus* Ruthe; 86. *Lipolexis gracilis* Foerster; 87. *Lysiphlebus desertorum* Starý; 88. *Lysiphlebus fabarum* (Marshall); 89. *Pauesia antennata* (Mukerji); 90. *Pauesia hazratbalensis* Bhagat; 91. *Praon exsoletum* (Nees); 92. *Praon rosaecola* Starý; 93. *Praon volucre* (Haliday); 94. *Praon yomenae* Takada; 95. *Trioxys complanatus* Quilis; 96. *Trioxys pallidus* (Haliday). High quality figures are available online.

**Figures 97–104.**
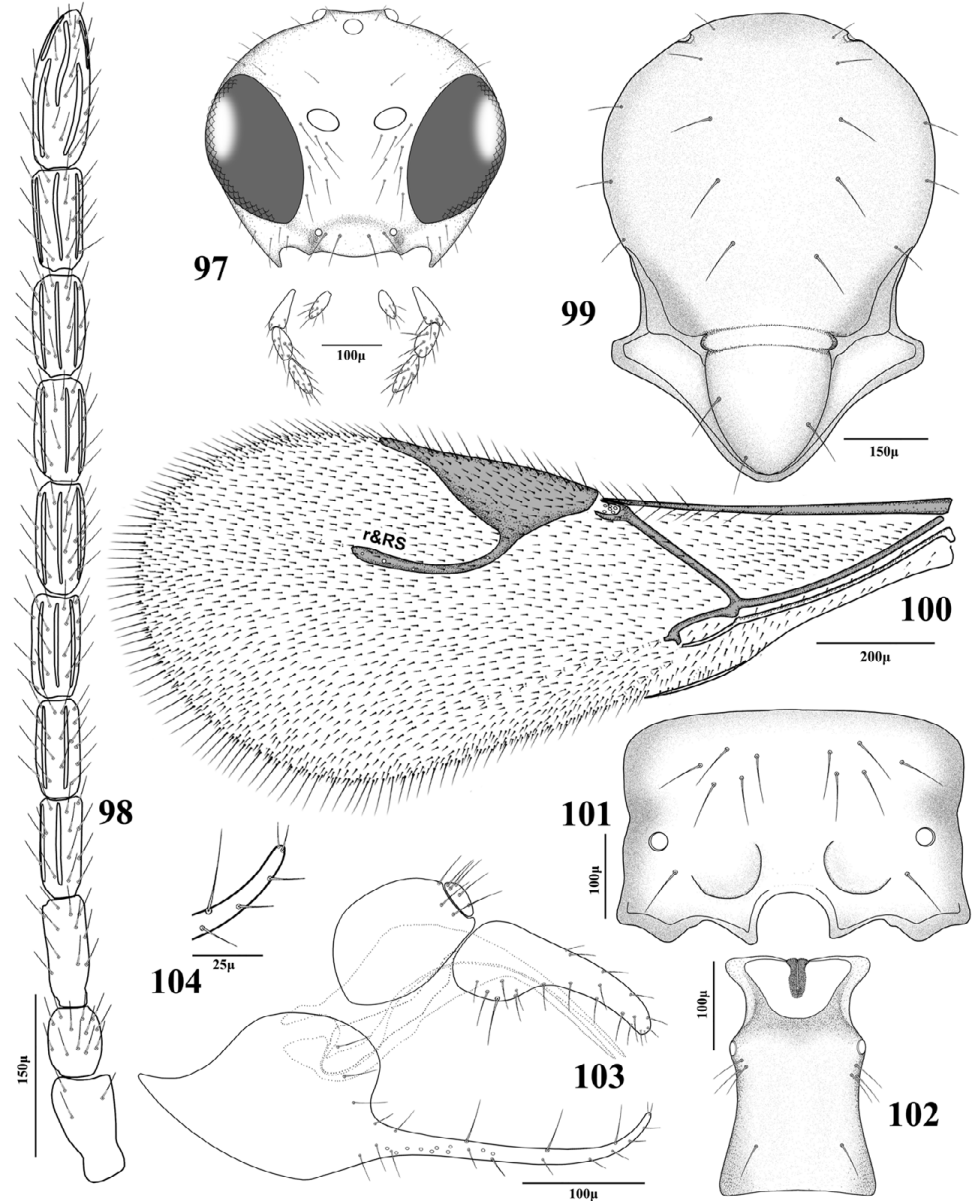
*Trioxys metacarpalis* Rakhshani and Starý sp. nov. (female). 97. Head and mouthparts; 98. Antenna; 99. Dorsal aspect of mesoscutum; 100. Forewing; 101. Dorsal aspect of propodeum; 102. Dorsal aspect of petiole; 103. Lateral aspect of genitalia; 104. Tip of prong. High quality figures are available online.
